# Mapping the digital determinants of health: a machine learning analysis of YouTube narratives on child undernutrition in Pakistan

**DOI:** 10.7189/jogh.16.04183

**Published:** 2026-06-12

**Authors:** Muhammad Shahid, Jiayi Song, Zaiba Ali, Hafiz Muhammad Naveed, Serhat Yuksel, Hasan Dincer

**Affiliations:** 1 College of Management, Shenzhen University, Shenzhen, Guangdong, China; 2 Department of Family Medicine, McGill University, Quebec, Montreal, Canada; 3 Department of Management, College of Business Administration, Princess Nourah bint Abdulrahman University, Riyadh, Saudi Arabia; 4 College of Management Science and Engineering, Shandong University of Finance and Economics, Jinan, Shandong, China; 5 School of Business, İstanbul Medipol University, Istanbul, Turkey

## Abstract

**Background:**

The role of social media in in shaping the public perception of health crises is growing, yet we know little about the contribution of platforms such as YouTube in shaping knowledge regarding child undernutrition. We aimed to explore the digital discussion of and sentiment around YouTube content on child undernutrition in Pakistan, where at least 38% of children are stunted (as per global rankings).

**Methods:**

We compiled a dataset of 1847 videos and 42 963 comments YouTube users that were uploaded between January 2010 and March 2024. We used Latent Dirichlet Allocation (LDA) topic modelling to analyse this data and identify narrative frames present in the content. We also used the Valence Aware Dictionary for sEntiment Reasoning (VADER) to analyses sentiment in the comments.

**Results:**

The analysis reveals six dominant narrative frames, where ‘policy failure’ made up 32% of the discourse, while just 5% addressed ‘solution-focused actions’. The VADER sentiment analysis supports this with a compound score of −0.72, suggesting an overall negative and highlighting considerable public concern about institutional responses. The data further suggests specific engagement patterns that point to a clear break between crisis-oriented and solution-oriented information.

**Conclusions:**

Algorithms on digital platforms amplify the narratives that serve as digital determinants of health, shaping public perceptions and reinforcing public distrust in institutional policy responses. It will not be enough to simply ‘get the message right’ if we seek to create effective health communication – we must, instead, be prepared to actively challenge harmful discourses, champion fair and locally relevant narratives, and engage for algorithmic transparency and public health-centred content curation from these platforms. These results provide an important evidence base for the reframing of nutrition communication initiatives in Pakistan and beyond.

Child undernutrition remains one of the most entrenched and inequitable global health problems that significantly impacts human capital, economic development, and intergenerational equity [1–4]. Almost two in five children under five years of age suffer from stunting, which is considered a marker of chronic malnutrition associated with increased mortality, impaired cognitive development, and decreased earning capacity in later life [5–8]. Despite national and international efforts aimed at addressing the issue of stunting, such as policy frameworks, donor-funded projects, and community-based approaches, progress has been slow globally, with tremendous geographical disparities specifically across Pakistan [9–11]. This suggests that child undernutrition is not just a biomedical problem [12], but also that, in Pakistan’s social and political context, it manifests through weak governance, income inequality, and negative public opinion [13,14].

Simultaneously, public life has been drastically digitalised, leading to changes in how health knowledge is produced, disseminated, and contested – for example, through fact-checking, debunking, opposing comments, and alternative narratives that challenge official or scientific claims [15]. These shifts led to social media platforms such as YouTube becoming critical public infrastructures, with narratives aggregated through algorithms emotionally charged and diffused in a manner that reflects and amplifies pre-existing power structures [16,17]. Here, we use the term ‘digital determinants of health’ (DDoHs) to refer to the conditions, affordances, and constraints shaped by digital infrastructures – *i.e.* platform architectures, algorithmic curation systems, user engagement mechanisms – that systematically shape access to health information in public discourse and policy environments [18–22]. Across health communication research, increasing insight is being gained into the importance of narrative framing – that is, the ways in which topics are presented and emotionally loaded within popular discourse – in influencing public understanding, policy salience, and community mobilisation [23–25]. However, they are ill understood in regard to the mechanisms where digital platforms algorithmically amplify certain narratives, while marginalising others, especially within low- and middle-income countries (LMICs) [26–28].

Interdisciplinary research on social media discourse has increasingly focused on critical health topics such as vaccination, COVID-19, HIV/AIDS, and mental health [29–32]. Nevertheless, considerable thematic, methodological, and contextual gaps remain. First, on thematic grounds, there is little research into the representation of chronic and structural problems like child undernutrition in digital spaces [33,34] – an issue embedded in structures of governance, inequality, and post-colonialism [35,36]. Second, the methodology applied in existing studies mainly either employs sentiment analyses or network analyses, while omitting topic mining approaches that would uncover latent narrative structures and their temporal trajectory relative to public sentiment – an omission pointed out by recent reviews on digital health research [37]. Third, the narrative framed by international and social media, as well as digital health research, has predominantly focused on western platforms and anglophone users overlooks the challenges of LMICs like Pakistan, potentially leading to a new form of neo-colonialism in health discourse [38,39].

Through his longitudinal computational analysis of YouTube content from Pakistan from 2010 to 2024, we want to fill this important gap in the research narrative on child undernutrition. We want to Latent Dirichlet Allocation (LDA) topic modelling and Valence Aware Dictionary for sEntiment Reasoning (VADER) sentiment analysis to explore temporal trends among prevailing narrative frames and their sentiments over the fourteen-year period, as the patterns observed during this time continue to shape contemporary conversations surrounding child undernutrition in Pakistan. We wanted to understand how algorithmic amplification can be biased in favour of particular narratives and to provide an operationalisation and empirical test of one important pathway through which DDoHs may operate in a high disease burden context. We use these DDoHs to contextualise our analysis, not because we are interested in measuring algorithmic systems directly, but because the framework clarifies how narrative patterns on digital platforms may matter for health policy. We examine one measurable aspect of these complex environments – public discourse on YouTube – but are aware that directly measuring algorithmic systems requires different types of data and methodologies. 

## METHODS

YouTube is one of the most used social media platforms, reaching about 79% of internet users [40]. It hosts a wide range of user-generated content, from news reports and expert analyses to personal narratives, making it a valuable lens through which to assess public narratives on health matters like child undernutrition.

### Data collection and corpus construction

We searched for videos about child undernutrition in Pakistan using keywords such as ‘child malnutrition Pakistan’, ‘stunting Pakistan’, ‘undernutrition Pakistan’, ‘child hunger Pakistan’, and ‘nutrition crisis Pakistan’. We then used Python and the Selenium framework retrieve the video URL, title, introduction, publisher, publishing time, comments, and the number of views and likes as part of our data collection process. To ensure search reproducibility and minimise personalisation bias, we conducted all searches using:

- incognito/private browsing mode with cleared cache and cookies before each search session;
- a VPN configured to Pakistan as the fixed geographic location to capture region-specific content;
- sorting by relevance (YouTube’s default algorithm) to reflect organic user exposure;
- standardised search timing conducted between 09:00 AM and 11:00 AM (Pakistan Standard Time) across three non-consecutive days.

We executed these search queries in English only. We excluded videos without comments, as they provided no discursive data for the sentiment or thematic analysis.

We filtered the following types of irrelevant comments manually: spam or promotional content; comments entirely in languages other than English; comments unrelated to child undernutrition topic (*e.g.* political discussions without health content); nonsensical or gibberish text. To ensure filtering reliability, two independent researchers coded a random sample of 500 comments (approximately 1.2% of corpus). Inter-rater reliability was assessed using Cohen’s *κ*, yielding substantial agreement (*κ* = 0.84). We resolved disagreements through discussion, after which a single researcher applied the final filtering protocol uniformly to maintain consistency.

**Figure 1:**
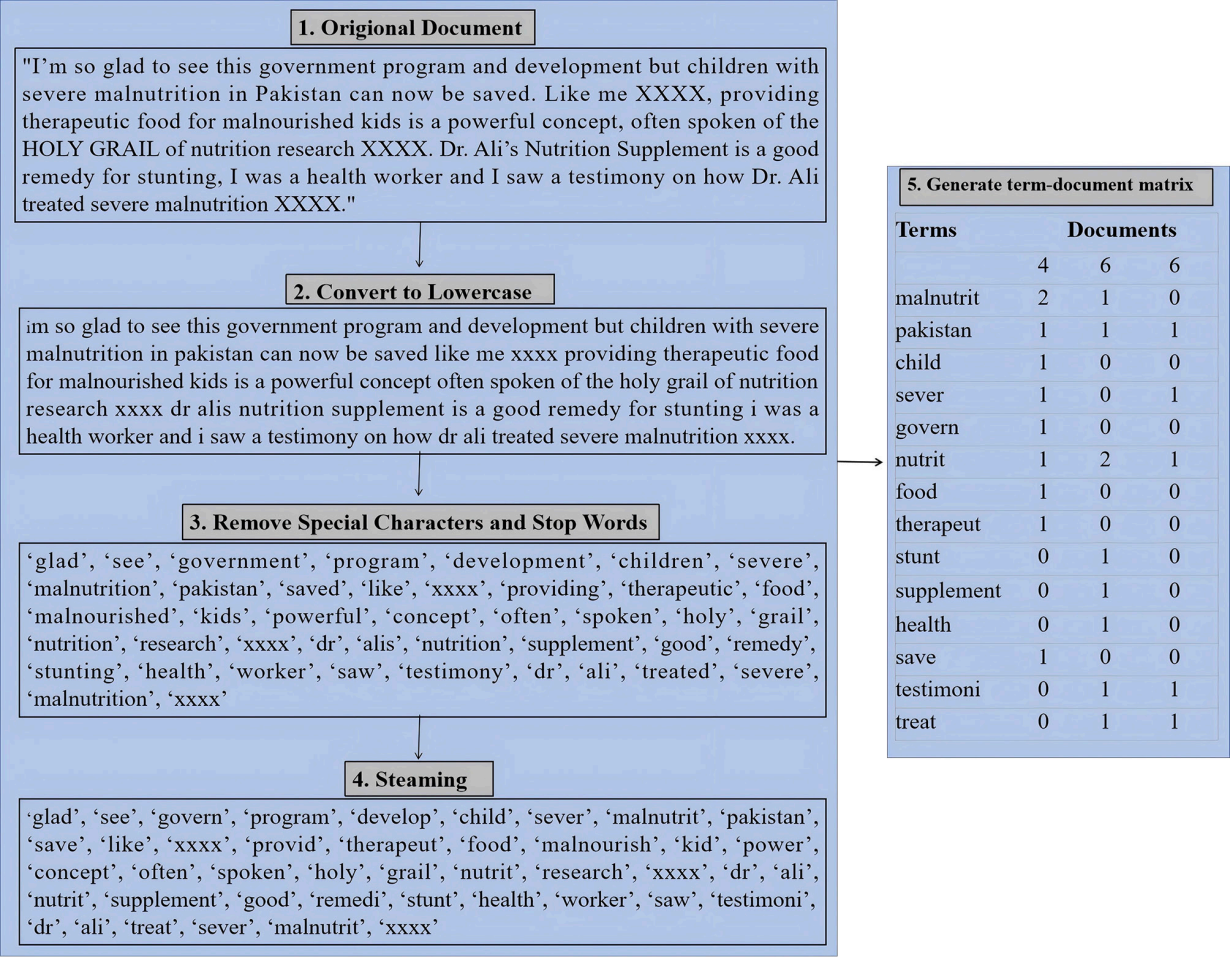
Text preprocessing and term-document matrix construction flowchart.

First, we converted all characters in the text to lowercase (**Figure 1**). Next, we used regular expressions to remove all numbers and non-alphabetic characters from the text. Then, we used the ‘word_tokenize’ method of the ‘NLTK’ library, version 3.8.1, to segment the pre-processed text and to remove English stop words, which helped reduce noise in the texts and improve the accuracy and efficiency of subsequent analysis. All analyses were performed using Python, version 3.9.7 (Python Software Foundation, Wilmington, Delaware, USA).

### Analytical framework

#### Descriptive/lexical analysis and LDA topic modelling

Preliminary analysis included temporal distribution of videos and comments, word frequency analysis, visualisation *via* word clouds and bar charts, and keyword co-occurrence network analysis using Gephi [41], an open-source network visualisation software.

The LDA is a probabilistic generative model widely used in text analysis. It identifies latent topic structures within texts by modelling the word distributions in document collections and capturing the core characteristics of different topics. To better interpret the discussions surrounding child undernutrition in Pakistan, we further integrated sentiment analysis techniques to explore the emotional distribution of major topics in YouTube comments. Our research design follows prior work that applies topic modelling to user-generated health commentary to track perceptions and sentiment dynamics on social platforms, alongside established practices for training and evaluating LDA models [42,43].

In particular, the LDA model has the following four steps in which the generative process is carried out. First, it assumes that the word distribution of each topic *δk* follows a Dirichlet distribution with parameter *β* that determines the smoothness of word distribution:



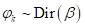



Then, the topic distribution *θ_d_* of each document is supposed to be a Dirichlet distribution parameterised by α which is a concentration of topic distribution:



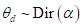



In the third step, a topic *Z_d,n_* is assigned to each word in the document by sampling from a multinomial distribution based on *θ_d_*, and then a specific word *w_d,n_* is generated from the word distribution *φ_Zd,n_* corresponding to the assigned topic:



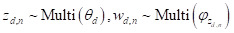



Lastly, the LDA model infers the topic by computing the marginal probability of the document collection through a joint probability formula:



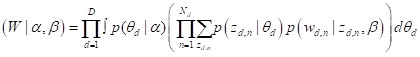



In practice, the performance of the LDA model is closely tied to three key parameters: the topic concentration parameter *α*, the word smoothing parameter *β*, and the number of topics *K*. Because of the inherent nature of a text corpus like YouTube comments, we also performed parameter tuning that assured salient topics arose from this randomised process.

One approach was to explore different configurations of the underlying model and examine well-defined, semantically separate topics [44]. This method bought in *K* flexibility, and used it at 4, 5, 6... up to 10. The parameters of the model were specified as follows:

- *α* = 0.1 (symmetric), resulted optimally from choices within (0.01, 0.05, 0.1, 0.5, and also shows slight benefits at value = 1) in terms of improving topic sparsity;
- *β* = 0.01 (symmetric), calibration beta forms for to ensure proper distribution of words in topic;
- set a minimum threshold for document frequency to eliminate noise in the analysis (*e.g.* ignore terms that occurred in fewer than five documents);
- document frequency threshold, *i.e.* terms that occurred in over 80% of documents were removed as corpus-specific stop words;
- for robustness checks, we ran models with five random seeds and checked convergence, obtaining less than 3% difference in topic coherence scores between runs.

The coherence scores were computed using *K* values ranging from 4 to 10, by employing the ‘C_v’ metric.

- *K* = 4: 0.412
- *K* = 5: 0.447
- *K* = 6: 0.523 (optimal)
- *K* = 7: 0.498
- *K* = 8: 0.471
- *K* = 9: 0.443
- *K* = 10: 0.421

The failure in consistency beyond a *K* of 6 indicates the optimum topic number choice. To check the conceptual clarity and distinctiveness of the developed topics, we supplemented this quantitative assessment through manual checks of high-probability terms and comments within the samples.

We obtained the highest coherence score on a six-topic solution, which was successful at preserving non-overlapping conceptual clusters. Models with less topics (*K* ≤ 6) were frequently confusing policy and socio-cultural themes, while those with many topics (*K* > 6) yielded fragmented and semantically weak themes. Hence, a *K* of 6 was chosen as the best number of subjects of this data.

To further justify this decision, we checked the stability of the model by holding out perplexity on a split 80/20 train-test. The perplexity curve was inflecting at the 5–6 range of topics, which corresponds to the likely maximum in the coherence analysis. This overlap in the quantitative indicators supported the choice of *K* of 6 as the arrangement that best balances thematic distinctiveness with model parsimony to be used in further analysis. Besides perplexity, we measured topic coherence measure to determine the interpretability of the model. This check results in a value between 0 and 1, denoting the level of semantic and statistical integrity of words in a topic; the higher the score, the more semantically consistent is the topic. The highest value of this measure of coherence allowed us to identify the most appropriate number of themes for the corpus.

For the temporal analysis of topic trends and the resulting development of sentiment, we averaged the topic proportions and normalised them monthly, allowing us to determine the monthly popularity index of each topic:



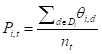



The signal *i* represents the topic identifier *t*, where *t* is a specific time interval, *d* a particular document (or comment), *n_t_* is the total number of documents gathered in time period *t*, and *θ_i,d_* is the contribution made by topic *i* to document *d*. The longitudinal outlook on the development of the discourse is made available to the analysis through the dynamic surveillance of this popularity measure. This enables a holistic analysis of how collective attention to child undernutrition changes over time and how sentiment patterns evolve.

#### Topic labelling validation

To systematically verify topic naming, two researchers conducted an exploratory data analysis by reviewing the 20 most common terms for each topic and a random subsample of 50 comments per topic. Each researcher independently proposed theme names, after which discrepancies were discussed until a consensus was reached. A third researcher performed a face validity check on the final labels, which were further validated against existing literature on nutrition discourse in Pakistan.

To assess consensus on the thematic labels, we independently coded 100 randomly sampled comments into six final themes. Inter-rater agreement was substantial (Cohen’s *κ* = 0.81), indicating an excellent degree of reliability for the labelling process.

### Sentiment analysis

We operationalised the sentiment polarity of the comments in English using VADER, a lexicon- and rule-based model for detecting sentiment of short, informal text [45]. This allowed for more sophisticated detection of sentiment in the sometimes broken and informal language used on the internet. The VADER analysis returns a compound score of sentiment on a scale from −1 for the most negative to +1 for the most positive sentiment.

We conducted a manual validation sub-study to evaluate how effectively VADER could analyse Pakistani English social media text. Using stratified random sampling, two researchers independently coded sentiment polarity (negative, neutral, and positive) on 500 comments, ensuring proportional representation of the three sentiment types across all six topic theme. We then compared the agreement of VADER’s classifications to that of the human coding, arriving at an overall accuracy of 78.3%, precision for negative sentiment of 0.81, recall for negative sentiment of 0.76, and a Cohen’s *κ* of 0.71 (substantial agreement).

Compound score thresholds for classification were <−0.05 (negative), −0.05 to 0.05 (neutral), and >0.05 (positive), following VADER documentation conventions [45]. As a sensitivity check, we compared VADER results with TextBlob sentiment analysis [46] on a 1000-comment subsample; Spearman's rank correlation between the two tools was a ρ of 0.68 (P < 0.001), indicating reasonable convergent validity.

To reduce the risk that inferences depend on a particular slice of the corpus, we explored whether aggregate polarity remained consistent across major time windows and across the LDA-derived topic clusters.

### Ethical considerations

User-level demographic characteristics are limited in the YouTube commenting interface and no *post hoc* generalisations were inferred. To avoid the misclassification of a cross-cultural corpus, all analyses were conducted on the full dataset without demographic stratification, as YouTube comments do not contain user gender or other identifiable demographic information. All analyses aligned with ethical standards for digital medical research [47] and used only publicly available data that does not contain personal identifiable information.

## RESULTS

We retrieved 2315 videos featuring 582 000 comments published between January 2010 and April 2024. After manual filtering, we retained 1847 videos and 42 963 comments remaining for our analysis. The temporal trend showed a dramatic increase in public involvement, as 77.2% (n = 1425) of the videos and 83.7% (n = 35 931) of comments were posted after 2018. Peak activity occurred during 2019-2021, correlating with major national nutritional surveys and flood-related humanitarian crises in Pakistan (Table S1 in the **Online Supplementary Document**).

### Word cloud analysis

**Figure 2:**
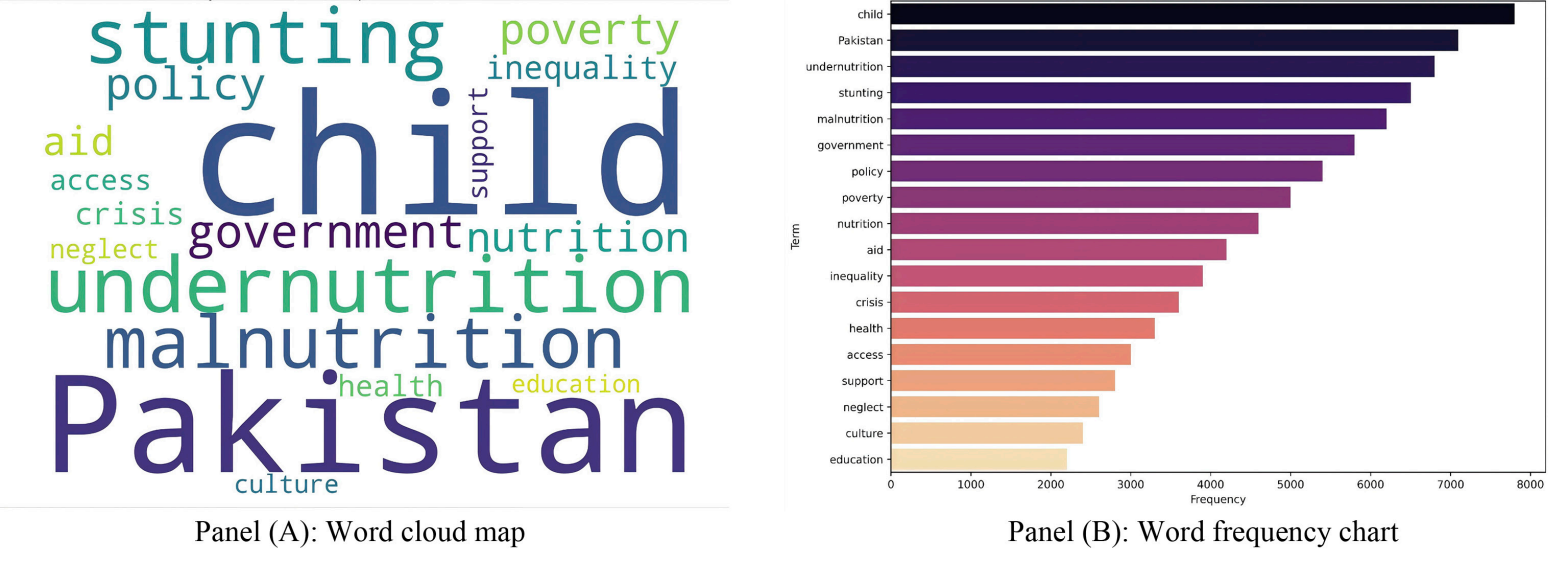
Word cloud map (**Panel A**) and word frequency chart (**Panel B**) for child malnutrition terms.

The dominance of ‘Child’ and ‘Pakistan’ in a word cloud visualisation represents the core element of what we are investigating, while terms like ‘stunting’ and ‘malnutrition’ emphasise key medical issues relevant to the situation. The words ‘poverty’ and ‘crisis’ highlight the economic and structural dimensions that place the issues in context. Terms such as ‘education’, ‘health’, and ‘culture’ appear less frequently, but are nonetheless central to the overall discourse (**Figure 2**, Panel A).

### Keyword analysis

Keyword analysis (**Figure 2**, Panel B) shows that the most frequent mention word was ‘child’, with 7800 mentions. This aligns with the fact that 51% of undernourished children in all ages are from Pakistan. Hence, when discussing Pakistan’s crisis of undernutrition (focusing on acute malnutrition), it is important to consider child welfare more broadly and ensure that the special status of children as one of the most vulnerable groups is recognised. ‘Government’ appeared 6190 times and ‘Pakistan’ appeared 7150 times. While terms such as ‘economic geography’, ‘institutional geography’, and ‘geographic health’ are hardly ever mentioned in their own right, repeated references to ‘Pakistan’ and the ‘government’ reinforce both the geographic and institution dimension of this challenge and reflect prevailing narratives about social accountability for the undernutrition problem.

The words ‘nutrition’ and “stunting’ appeared 5870 and 4230 times, respectively, pointing to the importance of these two issues among others related to health, growth indicators, and food security, such as ‘poverty’, ‘health’, and ‘malnutrition’. The frequent mention of ‘poverty’ (4600 times) in our dataset links undernutrition to socioeconomic status, suggesting that public discourse views this as an issue outside immediate individual action. Similarly, the 3800 mentions of ‘aid’ and references to non-governmental organisations and the United Nations Children’s Fund (United Nations Children’s Fund) imply that humanitarian agencies and international organisations are perceived as key actors in solving the problem.

Lower frequency terms included ‘policy’ (mentioned 4800 times), ‘aid’ (mentioned 3800 times), ‘inequality’ (mentioned 3500 mentions), ‘access’ (mentioned 2500 times), ‘culture’ (mentioned 1800 times), and ‘education’ (mentioned 1600 times). A more fine-grained examination of these terms in their contexts could make clearer the ways in which they are applied and mean within differing narrative frames.

These six themes represent an analytic tool to organise the YouTube discourse on child undernutrition in Pakistan: the affected population (child), national context (Pakistan), state responsibility (government), the health condition itself (nutrition, stunting, malnutrition); structural drivers of undernutrition(mid-level-causes/determinants), and external actors/responders (NGOs, UNICEF). The lexicon presents the issue as a national governance crisis with strong humanitarian and aid dimensions, rather than a mere medical or individual behavioural one.

### Semantic analysis

**Figure 3:**
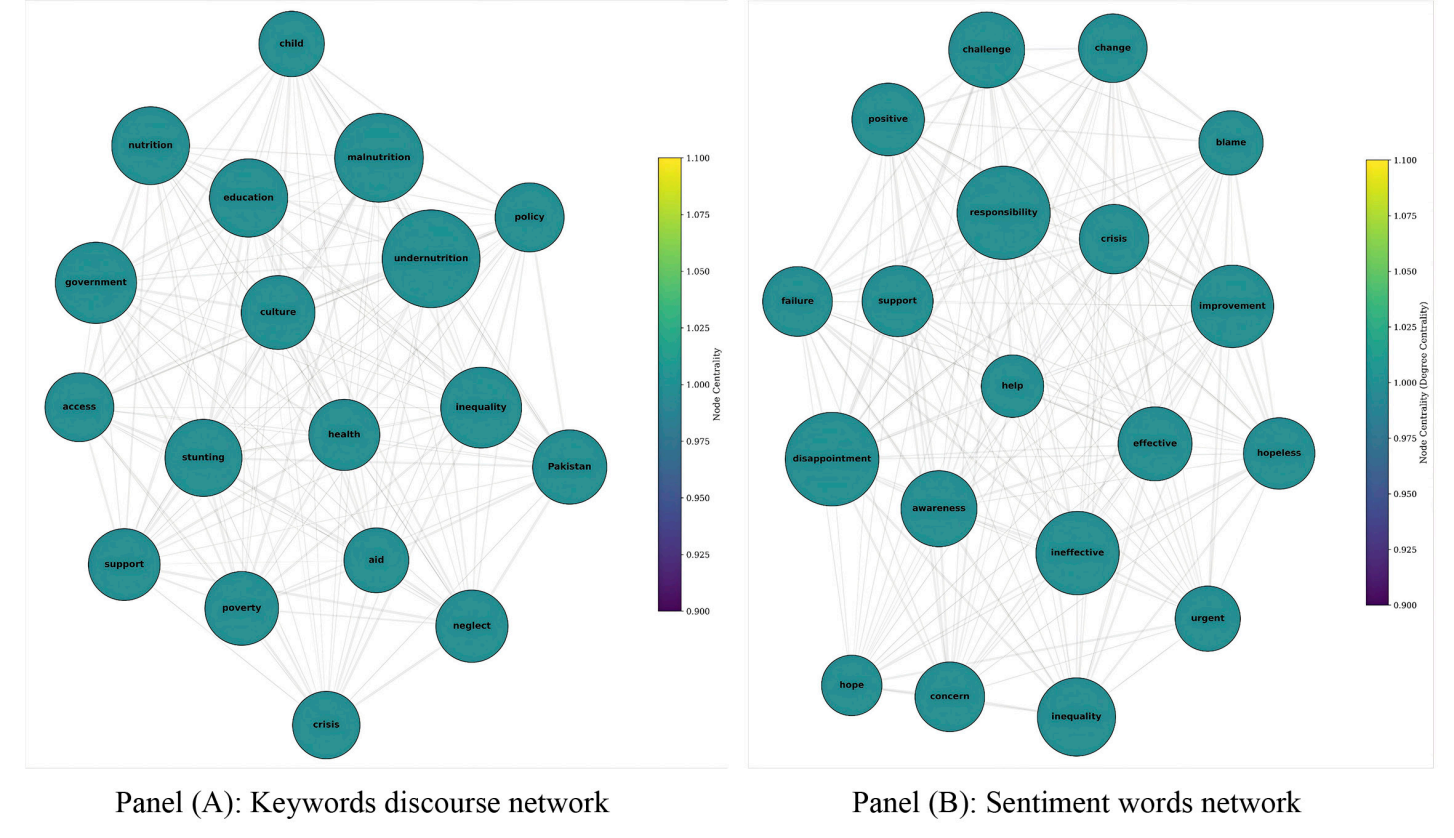
High-frequency keywords (**Panel A**) and sentiment words co-occurrence network (**Panel B**) in child undernutrition discourse.

We created a co-occurrence network to represent the relationships between keywords and their interconnectedness in the discourse regarding child undernutrition (**Figure 3**, Panel A). In the produced network diagram, ‘crisis’ occupies a central position in which many strong connections converge, suggesting it to be the core axis connecting various mainstream topics. The keywords ‘policy’ (mentioned 4800 times) and ‘neglect’ (mentioned 2000 times) are semantically related to ‘crisis’, together presenting a narrative of systemic governance failure and lack of oversight. The node ‘children’ is directly connected to both ‘nutrition’ and ‘health’, which are central concepts in the discourse. The strong association of the term with both ‘child’ and ‘nutrition’ provides an important point of reference for the specificity of content, while its relation to the term ‘policy’ implies that nutritional outcomes are assessed in contemporary public discourses through a lens of (non)governmental action. The keyword ‘access’ (mentioned 2500 times) is also related to this core, connecting the discourse to issues of resource allocation and equity. The keyword ‘culture’ (mentioned 1800 times) is part of the network, but less central, representing a contextual or contributing element in the discourse. The keyword ‘Pakistan’ (mentioned 6800 times) provides national localisation of the discussion.

The co-occurrence network (**Figure 3**) shows different line widths between nodes to represent the strength of co-occurrence. Stronger associations are detected between core keywords such as ‘crisis’ (mentioned 3200 times) and ‘failure’ (a key term in theme 0), revealing that these two concepts have a higher probability of occurring together. Conversely, words such as ‘support’ (mentioned 2200 times) have weaker connections and are more peripheral in the network

### Sentiment analysis

Our analysis shows that about 72% of comments in our sample were negative, 10% were positive, and 18% were neutral (**Table 1**). Criticism of government performance may reflect engaged citizenship and demand for accountability, rather than hopelessness or fatalism. While VADER classifies such language as negative, we acknowledge that negative sentiment toward institutions does not necessarily translate into a negative outlook on the possibility of solutions. However, the overwhelming predominance of negatively valanced language, combined with the minimal presence of solution-oriented narratives, suggests that the discursive environment provides limited exposure to constructive content – regardless of whether individual commenters maintain private optimism. To encourage a more positive discourse, policymakers should better understand public sentiment on child undernutrition and strategically communicate evidence of effective solutions and local successes. Neutral comments (18% of the total) indicate that some readers adopt a more factual approach, without expressing intense positive or negative feelings, their focus being on description or information. While sentiment strength may differ by topic (*e.g.* policy-centric threads display the most negative language in the aggregate), the general direction of the aggregate signal is fairly consistent, suggesting it to be a common occurrence on YouTube.

**Table 1:** Keywords and corresponding discourse proportions for the six themes (1847 videos)

Theme	Theme Categories	% of discourse	Keywords
Theme 0	Policy failures and government inaction	32.1	government, corruption, ineffective, neglect, bureaucracy, failure, promises, accountability, system, broken
Theme 1	Aid dependency and international intervention	20.0	donors, dependency, UNICEF, foreign, NGO, agenda, funds, external, assistance, humanitarian
Theme 2	Socioeconomic determinants and structural barriers	18.0	poverty, inequality, inflation, floods, climate, helpless, unemployment, food security, economic, crisis
Theme 3	Systemic neglect and geographic disparities	13.0	rural, infrastructure, access, facilities, hospitals, remote, disparity, services, healthcare, distance
Theme 4	Cultural factors and behavioral practices	12.0	tradition, mother, education, breastfeeding, women, norms, awareness, practices, beliefs, family
Theme 5	Solution-based actions and community responses	5.0	community, program, intervention, education, local, success, innovation, hope, effective, change

NGO – non-governmental organisation, UNICEF – United Nations’ Children’s Fund

The network visualisation (**Figure 3**, Panel B) shows the clustered emotional language in the discourse. Negative sentiment terms like ‘failure’, ‘crisis’, and ‘neglect’ are clustered as a dense block that have strong correlation with discussions around ‘policy’ (mentioned 4800 times). Positive sentiment terms such as ‘hope’, ‘support’, and ‘improvement’ build a smaller cluster that is linguistically more homogeneous (although also less coherent) and frequently occur in community or action-related contexts. From this, we see that discourse is polarised in terms of sentiment: negativity dominates in the inner part, while positivity stays only at margins.

### LDA modelling outcome

**Figure 4:**
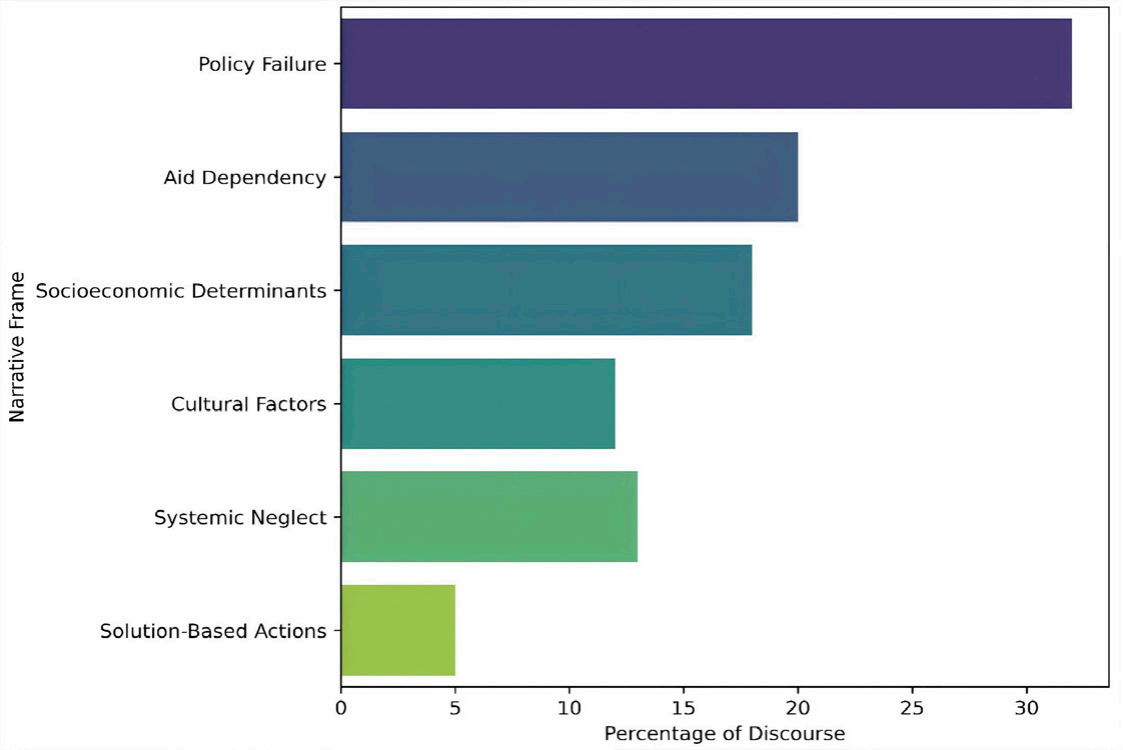
Narrative frame distribution across the YouTube corpus on child undernutrition in Pakistan.

The LDA thematic model analysis demonstrates the complexity of public attention regarding child undernutrition in Pakistan (**Figure 4**, **Table 2**, **Table 3**). Theme 0 (‘policy failures’) dominate the discussion, accounting for 32.1% of all discourse and expressing the most negative tone (−0.72). The high proportion (32.1%) of the ‘policy failures’ frame suggests substantial public attention to corruption in governance. In contrast, theme 5 (‘solution-based actions’) has a very high positive sentiment (+0.81), but comprises only 5.0% of the discourse. Theme 1 (‘aid dependency’) makes up 20.0% of the discourse, and theme 2 (‘socioeconomic determinants’) makes up 18.0%. The former shows mild negative sentiment (−0.18), while the latter shows strong negative sentiment (−0.65). Both frames show moderate engagement, indicating public concern about external aid mechanisms and structural poverty. Theme 3 (‘systemic neglect and geographic disparities’) and theme 4 (‘cultural factors and behavioural practices’) together account for about a quarter of the discourse with respective sentiment scores of −0.55 and −0.31, respectively. Overall, themes 0–4 represent 95% of the corpus, while theme 5 (‘solution-based actions’) accounts for the remaining 5%.

**Table 2:** Exploring the narratives: sentiment analysis of YouTube videos on child undernutrition in Pakistan

Sentiment classifications	Numerical value	Representative terms
Negative	72%	Failure, crisis, neglect, corruption, hopeless, shame, ineffective, inequality
Neutral	18%	Government, policy, nutrition, data, report, program, access, information
Positive	10%	Hope, support, community, effective, improvement, help, success

**Table 3:** Thematic analysis matrix: narrative frames with sentiment scores, engagement rates, and policy implications

Frame	Sentiment score	Engagement rate	Policy implications
Policy failures	−0.72	High	Need transparent reporting and accountability mechanisms
Aid dependency	−0.18	Medium	Localise success stories and promote domestic ownership
Socioeconomic determinants	−0.65	Medium	Link nutrition programmes to social protection systems
Systemic neglect	−0.55	Medium	Integrate nutrition services with WASH and primary healthcare
Cultural factors	−0.31	Low	Engage religious and community leaders in awareness campaigns
Solution-based actions	0.81	Low	Amplify positive content and scale proven local interventions

WASH – water, sanitation, and hygiene

## DISCUSSION

We used LDA topic modeling, sentiment analysis, and temporal tracking to examine public attention to child undernutrition in Pakistan, as reflected in YouTube content. Our results shed light on the relationship between user-generated digital conversations and the political landscape in Pakistan. Our findings show the emergence of a digitally mediated public sphere in which conversations occur about political breakdown and systems failing people, with little opportunity for transformative dialogue on the edges. In this context, approximately 95% of the YouTube content and comments in our dataset focused on crises and problems, while only 5% focused on solutions. These differences suggest varied modes of narrative engagement across platforms, raising questions for future research about how platform structures shape health discourse. Although our data do not directly measure YouTube’s internal recommendation systems, our findings reveal a consistent pattern where negative and crisis-oriented narratives are more prevalent than solution-focused content. However, these trends are not conclusive proof of a narrative selection influenced by platform limitations. Finally, while our findings reveal narrative imbalances on YouTube, we cannot attribute these imbalances to algorithmic amplification. Doing so would require experimental designs or access to proprietary platform data, which is beyond the scope of this observational study. Future research should directly examine the causal role of recommendation algorithms in shaping health narratives.

These results echo some trends in digital health discourse found in other LMICs, while also showing unique divergences. A study from India highlighted the relevance of state-level programmatic efforts to enhance child nutrition [35], while studies from sub-Saharan Africa indicated the importance of community resilience and using local language content during crises [36]. In Pakistan, though, the health discourse is characterised by an unusually intense focus on governance failures which is closely tied to the country-specific political economy and the realities of recent humanitarian crises. 

The frequent mention of the term ‘child’ (7800 times in our dataset) aligns with national data showing that 51% of undernourished children in all ages are from Pakistan [5]. This highlights the importance of recognising children as one of the most vulnerable groups when discussing Pakistan's undernutrition crisis.

The ‘policy failure’ narrative accounts for around one third of all discourse, demonstrating a deep-rooted cynicism towards state institutions. Research on the digital ecology of Pakistan has indicated that content related to news and political affairs is mostly negative and conflict-based, as creators strategically use such framing to gain audience attention or viewership [48,49]. The collocation between ‘government’ and ‘failure’ observed in our data captures public discourse associating state institutions with inadequate responses to child undernutrition. Our co-occurrence network analysis reveals that public discourse on YouTube positions child undernutrition not merely as a health challenge but as a multidimensional crisis embedded with sentiments of policy failure and institutional neglect in the Pakistan context. This environment creates a feedback loop in which governance-related grievances are amplified by algorithms that privilege emotionally-relevant content [50,51]. One study describes this phenomenon as ‘algorithmically induced cynicism’, in which digital platforms amplify distrust and stymie serious discussions of policy [52].

The theme of ‘aid dependency and the international response’ highlights some of the geopolitical aspects present in digital health narratives. While the narrative in India or China has largely revolved around the ideas of combating malnutrition [53,54], for some parts of Pakistani society, articulating misfortune through humanitarian discourse tends to dominate the narrative, rather than a focus on local solutions or resilience. This external perspective may reduce the visibility of successes at the local level [53]. The perspective of international donors and humanitarian organisations may reduce the visibility of local successes in Pakistan [53]. The ‘aid dependency’ framing risks reinforcing colonial discourses of dependency [52], which are often used to subsume local agency [53]. As a result, it is essential to promote locally produced content in Urdu and regional languages promote locally produced content in Urdu and regional languages homegrown content that showcases successes of Pakistani-led efforts. In sum, the frequent mention of terms such as ‘policy’, ‘aid’, and ‘inequality’ signals the structural and political dimensions of the undernutrition crisis as perceived by YouTube users

The theme ‘solution-oriented narratives’ makes up only 5% of the conversations. This low proportion may reflect multiple factors, including a lack of local solutions, lower production quality of solution-focused content, or platform visibility dynamics – though our data cannot determine which factor is the most responsible. The media and digital literacy and content creation capacity are unevenly distributed in Pakistan [55], meaning that positive local narratives often lack the professional production quality – such as high-resolution video, clear audio, professional editing, and search engine optimisation – required to compete with crisis-oriented coverage. Algorithmic biases that prioritise high-engagement content also further marginalise constructive narratives [55]. The presence of keywords such as ‘access’, ‘culture’, and ‘education’ in our dataset suggests that YouTube users associate potential solutions with infrastructural and behavioural factors. When failure narratives dominate digital spaces, public confidence in progress can decline, thus weakening support for investments in nutrition [56].

Using temporal analysis, we observed a significant increase in both volume and negative sentiment of the discourse during national emergencies (like floods) and political transitions, reinforcing the notion that digital narratives are overly responsive to sociopolitical events [57]. The negative sentiment observed in our data should be interpreted with caution. Criticism of government performance may reflect engaged citizenship and demand for accountability rather than hopelessness or fatalism. Identifying moments of increased public attention during crises could provide opportunities to align health communication strategies with ongoing public concerns and information-seeking behaviour [58]. The predominance of negative sentiment in our data may reflect public awareness of Pakistan's chronic malnutrition crisis and could evoke sympathy for affected children and families, though our data cannot directly measure emotional responses.

Any optimism expressed in the 10% of positive comments may reflect local community actions, regional efforts, or hopeful visions for effective solutions, though our data cannot directly measure the basis for this sentiment.

Our results suggest that a shift from information dissemination to narrative engagement is critical for health communication practice. In order for local health advocates and community leaders to be able to create content that directly competes against the platform economy, the next step is improving their digital literacy in these regards (data up). Moreover, engaging in dialogues with platform-corporations to address context-related algorithms may help promote solution-cantered health information [59].

The flood of crisis narratives invites an important question: is this tendency a sign of commitment to realism, or excessive pessimism? Considering that 40% of Pakistani children are stunted [5], one could argue that it is quite right to frame things negatively. However, a narrative that dwells entirely on failures, while ignoring possibilities of fixing everything undermines synergistic potency. The content focusing on solutions is only 5% of the conversation, revealing a systematic marginalisation of constructive conversations.

The interactivity of problems and solutions in relation to child undernutrition indicates that the digital environment of neglect dominates the narrative with narratives surrounding failure predominating over potential solutions. When dealing with this issue, parallel approaches should be prescribed with not just specific nutritional interventions, but wider systems-wide strategies to shift the existing narrative ecosystem in line with these places [60,61]. Such initiatives would enable Pakistan to present its own narratives of struggle and resilience, allowing for a more nuanced and constructive dialogue.

In sum, the contributions of this research are three-fold in the context of Pakistan. First, it presents one of the first empirical examinations in how child undernutrition – a structural issue with deep political roots – is narratively constructed on a major digital platform in a high-burden LMIC. By contextualising these platform algorithms within the DDoH framework, we offer a model for future research to examine digital health discourse in similar environments. Second, methodologically, we demonstrate the usefulness of integrated computational techniques (LDA combined with sentiment and temporal analysis) to detect not just topic prevalence (*i.e.* how often each theme appears), but also framing and emotive content over time. This method provides a model for health communication researchers to analyses intricate, longitudinal digital discourses beyond rudimentary metrics. Third, in the context of policy and decision-making, our digital narrative landscape mapping provides an evidence-based diagnosis for ‘public perception’ of child undernutrition in Pakistan. It uncovers not just dominant framings of blame and helplessness, but also marginalised stories that focus on local responses. This data is critical for designing culturally specific, digitally informed communication strategies to combat fatalism, enhance community agency, and support policy advocacy efforts in Pakistan and other settings. Lastly, we argue that addressing child undernutrition in the digital age requires consideration of digital determinants of health alongside traditional biological and social determinants. On the whole, we seek to facilitate research and decision-making towards a better, fairer health communication and policy interventions by identifying how YouTube influences public discourse in Pakistan.

### Limitations

There are several important limitations that should be considered within the context of this study. First, the analysis is limited to English-language content and does not include discussions in Urdu or other regional languages. This exclusion may bias findings toward narratives from more urban, educated, and higher socioeconomic circles and exclude community-level discourse and culturally specific framings. Future research should employ multilingual natural language processing methods to gain the broadest picture of the discursive landscape. In this context, the VADER may not sufficiently capture culturally specific expressions of sentiment in Pakistani English. Second, selecting only videos with comments might have resulted in an over-representation of content found on YouTube, where the level of engagement was higher and that necessarily had a more controversial nature. We opted for this methodological choice because we required discursive data, *i.e.* comments, to perform sentiment and thematic analyses of public discourse, even though it would skew our results towards divisive narratives and away from informative content that tends to generate lower emotion. Comparative future research based on commented and non-commented video content would be valuable in the future to identify systematic differences between these sources. Third, as noted above, we have no access to YouTube's proprietary recommendation algorithms and cannot make causal claims about how this platform shapes narratives. Fourth, despite the longitudinal nature of our computational analysis, we cannot draw causal inference on whether exposure to narratives causes subsequent changes in public opinion or policy attention with this cross-sectional observational study. Fifth, the lack of demographic information about those who viewed and commented on our narratives limits the scope of our ability to explore how narrative reception might be distributed along socioeconomic, geographic, or gender-based lines in Pakistan. Sixth, while our findings reveal narrative imbalances on YouTube, we cannot attribute these imbalances to algorithmic amplification. Doing so would require experimental designs or access to proprietary platform data, which is beyond the scope of this observational study. Future research should directly examine the causal role of recommendation algorithms in shaping health narratives. Seventh, cross-sectional data do not permit an assessment of causal direction. The dominance of negative narratives could be a product of platform effects, or it might reflect existing public sentiment. Most likely there is a feedback effect, where public worry encourages engagement, which in turn may prompt algorithms to boost this content further. Future research should utilise experimental designs to help clarify these dynamics. Finally, our quantitative analysis identifies keyword frequencies but does not examine how these terms are used in context. Future research could employ qualitative discourse analysis to explore the contextual meaning of keywords within different narrative frames.

### Recommendations and way forward

We derive several recommendations from our analysis. First, there is an urgent need to shift the existing nutrition communication strategies in Pakistan from a factual, information-based format to one emphasising strategic storytelling. This requires a refusal to champion narratives of victimhood and powerlessness, while actively promoting and sharing individual success stories that arise within communities. Sharing local success stories creates not only a sense of agency among individuals as active citizens, but also reinforces accountability mechanisms for state institutions. Second, public health institutes should consider building digital health observatories which would provide content analysis of the emerging narratives and attitudes about health over time. In doing so, they would be better able to time communication responses and align them with the changing dynamics of public discourse. Third, future research should examine how platform algorithms influence the visibility of health narratives, given that our study identifies narrative imbalances, but cannot determine their causes. Independent, transparent research on algorithmic effects – conducted without government or platform interference – is needed before any policy recommendations about content curation can be responsibly made. Moreover, promoting independent research to investigate the impact of algorithm could contribute to building a strong evidence-base that is really necessary for future discussions around its regulation.

## CONCLUSIONS

Most of the YouTube content explaining child undernutrition in Pakistan in our sample focuses on the failure of government institutions and the lack of attention from policymakers to the nutritional crisis. Only 5% of this content offers solutions, and only 10% of viewers’ comments show a positive sentiment. In our dataset, local stories of success appeared far less frequently than crisis-focused narratives. Our study contributes to the methodological literature on narratives in digital health by combining computational topic modelling with longitudinal sentiment analysis, thereby generating empirical understandings that can help us understand how such narratives shape digitally mediated pathways of health governance.

Our findings emphasise that in the digital age, controlling the narrative on health is just as important as being right about its science. We, therefore, propose the term ‘narrative justice’, understood in this context as ensuring fair representation of diverse voices, experiences, and solutions from communities in health narratives, particularly those that have historically been excluded from dominant national and global narratives. In the absence of such narrative justice, health equity will likely be undermined by digital discourse that routinely erases local agency and effective interventions.

## Additional material


Online Supplementary Document
