# Supplementary material for: Mapping the digital determinants of health: a machine learning analysis of YouTube narratives on child undernutrition in Pakistan
Source: J Glob Health. 2026 Jun 12;16:04183. doi: 10.7189/jogh.16.04183 (PMC13261325; doi:10.7189/jogh.16.04183)
Supplement: Online Supplementary Document [file jogh-16-04183-s001.pdf]

Supplement to: Shahid M, Song J, Ali Z, Naveed HM, Yuksel S, Dincer H. Mapping the digital determinants of health: a machine learning analysis of YouTube narratives on child undernutrition in Pakistan. J Glob Health. 2026;16:04183.

Supplementary Table S1. Temporal distribution of YouTube videos and comments on child undernutrition in Pakistan (2010–2024), n (%)*

Periods	Videos (n = 1847)	Comments (n = 42 693)
2010–2014	92 (5.0)	1102 (2.6)
2015–2018	310 (16.8)	5987 (13.9)
2019–2021	658 (35.6)	18 432 (42.9)
2022–2024	787 (42.6)	17 442 (40.6)

*The pre-processed text corpus comprised 4.1 million tokens with 12 883 unique terms after rigorous cleaning, enabling robust computational linguistic analysis.

Text S1. Explanation of authorship change statement

The authors have, after the submission of this manuscript, requested to add ZA as a co-author, who has inadvertently been omitted from the initial version. ZA has acquired funding support crucial for the conduct and publication of this research, especially in terms of financial support for the data processing and analysis. She has also contributed to the revisions of this paper. The authors provided manuscript versions to which ZA contributed, a revised funding statement, a signed authorship form, and individual confirmation via institutional e-mail addresses as proof of this change.
